# Ubiquitin ligase TRIM65 promotes colorectal cancer metastasis by targeting ARHGAP35 for protein degradation

**DOI:** 10.1038/s41388-019-0891-6

**Published:** 2019-07-22

**Authors:** Daici Chen, Yichen Li, Xiaowen Zhang, Haiyong Wu, Qian Wang, Jian Cai, Yanmei Cui, Huanliang Liu, Ping Lan, Jianping Wang, Zihuan Yang, Lei Wang

**Affiliations:** 1 Guangdong Provincial Key Laboratory of Colorectal and Pelvic Floor Diseases, The Sixth Affiliated Hospital, Sun Yat-sen University, Guangzhou, Guangdong China; 2 Guangdong Institute of Gastroenterology, Guangzhou, Guangdong China; 3 Department of Colorectal Surgery, The Sixth Affiliated Hospital, Sun Yat-sen University, Guangzhou, Guangdong China

**Keywords:** Colorectal cancer, Prognostic markers

## Abstract

Tripartite motif-containing protein 65 (TRIM65) is an E3 ubiquitin ligase and a critical regulator of a variety of cellular processes as well as tumor progression. Therefore, more substrates must be identified in the physiology or disease context. Here, we found that TRIM65 is upregulated and associated with poor survival in colorectal cancer (CRC). More specifically, high expression of TRIM65 is associated with CRC metastasis and recurrence. Ectopic overexpression of TRIM65 in CRC cell lines enhanced proliferation, invasion, and migration, while knockdown of TRIM65 expression had the opposite effects. Furthermore, we identified a new substrate of TRIM65, namely ARHGAP35, a Rho GTPase-activating protein (GAP) that is involved in polarized cell migration. Phenotypically, forced expression of TRIM65 induces increased production of migration-related structures, focal adhesions, and/or filopodia and enhances CRC metastasis to the liver or the lung in a mouse model. Mechanistic studies revealed that TRIM65 mediates ubiquitination of ARHGAP35, whose degradation leads to elevated Rho GTPase activity. In addition, we identified several phosphorylation sites on TRIM65. In sum, we reveal a novel TRIM65–GAP–Rho regulatory axis that modulates the actin cytoskeleton and the migration behavior of CRC cells, and the TRIM65–ARHGAP35 interaction might be a valuable therapeutic target in CRC.

## Introduction

Metastasis to distant organs is the most fatal factor for many solid organ tumors, including colorectal cancer (CRC). In CRC, the 5-year survival rate is over 90% for stage I disease, while it is below 10% when CRC develops into advanced stage IV disease with metastasis [1]. Targeted therapy is an essential part of the comprehensive regimen for treatment, when CRC progresses to advanced stages, especially with distant metastasis [2]. Therefore, it is continually necessary to elucidate the underlying mechanisms by which cancer cells migrate, invade, and cope with the microenvironment, with the aim of identifying biomarkers to predict the occurrence of metastasis and finding targets to halt these vital processes [3].

Cancer cells are motile, which gives them the ability to migrate and invade. This process involves remodeling of the cytoskeleton, particularly the actin and microtubule networks, whose activities and dynamics are controlled upstream by GTPases [4]. GTPases are molecular switches that are active in the GTP-bound form and inactive in the GDP-bound form, controlled by guanine nucleotide exchange factor and GTPase-activating protein (GAP), respectively [5]. Rho family GTPase, belonging to the Ras superfamily of GTPases, is a prototypic family of GTPases that has well-established functionalities in cytoskeleton regulation, with RhoA, Rac1, and Cdc42 as the three best-characterized members. Given the essentiality of cytoskeletal proteins in cancer cell migration, a therapeutic attempt to inhibit metastasis by targeting cytoskeletal proteins has shown promise, shedding light on this approach [6, 7]. More key players in the metastasis route have yet to be identified.

By sequence consensus, TRIM65 belongs to the tripartite motif family [8]. Originally identified as a gene with SNPs associated with cerebral white matter lesions [9–11], TRIM65 was later shown to be a cofactor for the regulation of microRNA function. TRIM65 regulates microRNA activity by ubiquitination of TNRC6 while establishing its E3 ubiquitin ligase activity [12]. Like other TRIM members that are involved in the immune response, TRIM65 also participates in the virus-induced innate immune response by ubiquitination of substrate proteins [13, 14]. In tumor malignancies, TRIM65 was first shown to harbor oncogenic activities in lung and liver cancers, and cancer-related TRIM65 substrates, including TP53 and AXIN1, had been reported [15, 16]. More recently, TRIM65 was reported to support bladder urothelial carcinoma cell progression by targeting ANXA2 for ubiquitination and degradation [17]. The significance of TRIM65 in CRC and particularly in metastasis, and the corresponding substrate, has yet to be determined.

We initially surveyed The Cancer Genome Atlas (TCGA) colorectal data for E3 ubiquitin ligase proteins with relatively low (FPKM < 10) yet differentially expressed and identified TRIM65 as an interesting one. We found that TRIM65 is upregulated and associated with poor survival in CRC. Interestingly, TRIM65 expression is associated with metastasis and relapse in patients. Ectopic overexpression of TRIM65 enhanced the proliferation, invasion, and migration of tumor cells in vitro. Using a quantitative proteomic approach (iTRAQ), we identified a new substrate of TRIM65, namely, ARHGAP35, a well-known Rho GAP that modulates the GTPase activity of Rho family proteins. We further confirmed the physical interaction between TRIM65 and ARHGAP35 by a proximity ligation assay (PLA). Degradation of the ARHGAP35 protein was induced through ubiquitination, which was enhanced by TRIM65, leading to elevated Rho GTPase activity. Phenotypically, forced expression of TRIM65 produced more migration-related structures, focal adhesions, and/or filopodia and enhanced CRC metastases to the liver or the lung in a xenograft or tail vein injection mouse model, respectively. In sum, we identified a novel TRIM65–GAP–Rho regulatory axis that modulates the actin cytoskeleton and the migration ability of cells in CRC, which may represent a regulatory process for cancer cell migration and metastasis in general. Furthermore, the TRIM65–ARHGAP35 interaction might be a valuable therapeutic target.

## Results

### TRIM65 expression is increased in CRC and correlated with poor outcome

**Fig. 1:**
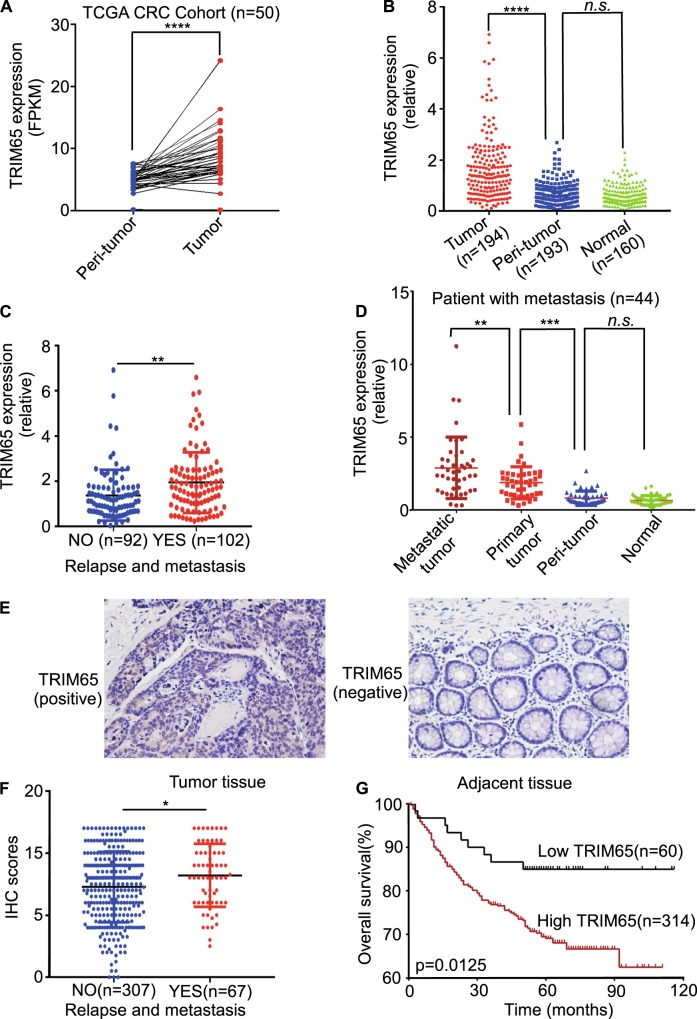
TRIM65 expression is increased in CRC and correlates with poor outcome. **a** mRNA expression level of TRIM65 in TCGA CRC samples compared with matched peritumor tissues. *n* = 50, ***p < 0.001, two-tailed Student’s *t*-test. **b** mRNA expression level of TRIM65 in CRC tissues, matched peritumor, and normal tissues of patients. ***p < 0.001, ns not significant (*p* > 0.05), two-tailed Student’s *t*-test. **c** mRNA expression level of TRIM65 in CRC tissues of patients with or without relapse and metastases. **p < 0.01, two-tailed Student’s *t*-test. **d** mRNA expression level of TRIM65 in CRC metastatic tumor, primary tumor, matched peritumor, and normal tissues of patients with relapse and metastasis. *n* = 44, **p < 0.01, ***p < 0.001, ns not significant (*p* > 0.05), two-tailed Student’s *t*-test. **e** Representative immunohistochemical (IHC) images of TRIM65 in a tissue microarray of paired samples of CRC (left panel) and adjacent normal colon tissues (right panel) of patients. **f** Statistics of IHC scores of TRIM65 expression in CRC tissue microarrays from patients with/without relapse and metastasis. The IHC scores of TRIM65 expression were assessed by two technicians and scores were averaged. *p < 0.05, two-tailed Student’s *t*-test. **g** Kaplan–Meier survival curves of overall survival duration based on TRIM65 expression in the CRC tissues of the cohort. Survival curves show that patients with low TRIM65 expression survived significantly longer than those with high TRIM65 expression. *p = 0.0125, log-rank test

We downloaded the TCGA mRNA expression data (level 3) for colon and rectal cancer. Among the 50 CRC tissues with matched normal tissues, the mRNA level for TRIM65 was significantly higher in the tumor tissue (Fig. 1a). By analyzing samples collected in our center, we also observed that TRIM65 mRNA was overexpressed in CRC tissues compared with adjacent normal tissues (Fig. 1b). Among the 194 patients, 102 patients were eventually diagnosed with relapse and metastasis. The TRIM65 mRNA level in these 102 patients was significantly higher than that in nonrelapse patients (Fig. 1c). Among these 102 patients, 44 patients had resected metastasized tumors available, along with primary tumor, peritumor (~2 cm from the tumor site), and normal (~5 cm from the tumor site) tissues. Strikingly, metastasized tumors expressed the highest level of TRIM65 (Fig. 1d).

The above cohort of 194 patients had uneven populations with stage I–IV disease. To investigate whether TRIM65 overexpression is correlated with the outcomes of CRC patients, another independent cohort of 374 patients from our center was recruited. The results from tissue microarray-based immunohistochemical (IHC) analysis showed that TRIM65 expression in almost all the CRC samples was positive. TRIM65 protein was positively stained in CRC glands but negatively stained in adjacent normal glands, and TRIM65 was mainly localized in the cytoplasm of epithelial cells (Fig. 1e). Similar to the TRIM65 mRNA expression results above, patients with relapse and metastasis expressed higher protein levels of TRIM65, based on IHC scores (Fig. 1f). ROC curve analysis did not succeed in finding a cutoff score that separates the patient cohort with significant difference of overall survival (OS). Instead, patients were best separated into two groups: high (scores > 4) or low (scores ≤ 4) TRIM65 expression. Kaplan–Meier analysis showed that a high level of TRIM65 was correlated with poor OS (Fig. 1g). High TRIM65 expression was also significantly associated with degree of differentiation (*p* = 0.015), pN status (*p* = 0.006), clinical stage (*p* = 0.035), and relapse and metastasis (*p* = 0.035) (Table 1). Univariate and multivariate analyses of different prognostic parameters revealed that TRIM65 expression was not an independent factor for overall survival (Table 2), but was a strong, significant indicator of relapse and metastasis.

**Table 1:** Correlation between expression of TRIM65 and clinicopathological features of CRC patients

Variable	Low TRIM65	High TRIM65	*p* value
Gender			0.389
Male	36 (60%)	170 (54.9%)	
Female	24 (40%)	145 (45.1)	
Median age			0.092
<59 years	27 (45.0%)	106 (33.7%)	
>59 years	33 (55.0%)	209 (66.3%)	
Differentiated degree			0.015
Poor (G1)	0 (0.0%)	19 (7.0%)	
Moderate (G2)	31 (60.8%)	191 (70%)	
High (G3)	20 (39.2%)	63 (23.1%)	
pT status			0.324
T1	6 (10.2%)	16 (5.2%)	
T2	15 (25.4%)	62 (20.1%)	
T3	34 (57.6%)	206 (65.2%)	
T4	4 (6.8%)	25 (7.9%)	
pN status			0.006
N0	52 (86.7%)	203 (65.9%)	
N1	6 (10.0%)	84 (27.3%)	
N2	2 (3.3%)	21 (6.8%)	
pM status			0.627
M0	56 (93.3%)	279 (90.3%)	
M1	4 (6.7%)	30 (9.7%)	
Clinical stage			0.035
I	20 (33.9%)	69 (24.2%)	
II	28 (47.5%)	125 (41.6%)	
III	7 (11.9%)	84 (24.7%)	
IV	4 (6.8%)	31 (9.5%)	
Relapse and metastasis			0.035
Yes	5 (8.3%)	62 (19.7%)	
No	55 (91.7%)	252 (80.3%)

**Table 2:** Univariate and multivariate analysis of different prognostic parameters for CRC patients

	Univariate analysis		Multivariate analysis	
Variable	HR (95%CI)	*p* value	HR (95% CI)	*p* value
Gender (male vs. female)	0.92 (0.63–1.35)	0.671	0.84 (0.54–1.30)	0.441
Age (<59 years vs.≥59 years)	1.17 (0.79–1.75)	0.44	1.27 (0.78–2.04)	0.336
Differentiated degree (G1 or G2 vs. G3)	0.59 (0.35–0.99)	0.047	1.09 (0.61–1.98)	0.766
pT status (T1 or T2 vs. T3 or T4)	7.49 (3.28–17.07)	<0.001***	2.87 (1.21–6.79)	0.017*
pN status (N0 vs. N1 or N2)	4.68 (3.16–6.94)	<0.001***	6.87 (1.95–24.13)	0.003**
pM status (M0 vs. M1)	11.68 (7.39–18.47)	<0.001***	10.72 (5.60–20.55)	<0.001***
Clinical stage (I or II vs. III or IV)	6.10 (4.05–9.19)	<0.001***	0.36 (0.09–1.41)	0.142
Relapse and metastasis (yes vs. no)	5.55 (3.78–8.14)	<0.001***	5.89 (3.76–9.22)	<0.001***
TRIM65 expression (low vs. high)	2.34 (1.18–4.63)	0.014*	1.24 (0.58–2.67)	0.584

*p < 0.05, **p < 0.01, ***p < 0.001

### TRM65 promotes the proliferation, migration, and invasion of CRC cells

**Fig. 2:**
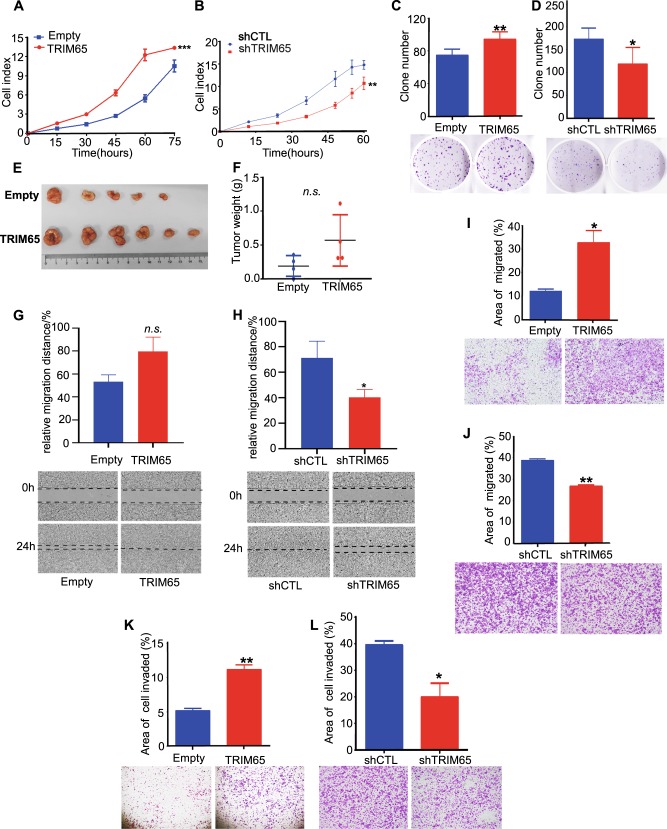
TRM65 promotes the proliferation, migration, and invasion of CRC cells. **a**, **b** Proliferation assay of KM12 CRC cells conducted by using IncuCyte. KM12 cells were stably transfected with *lenti-TRIM65* (termed as *TRIM65-*KM12) or *shTRIM65* vectors (termed as *shTRIM65*-KM12) in comparison with corresponding empty (*Empty-*KM12)/control (*shCTL*-KM12) vectors. ***p < 0.001, **p < 0.01, paired-sample *t*-test. **c**, **d** Images (lower panel) and statistical analysis (upper panel) of colony formation assays of *TRIM65-*KM12 and *shTRIM65*-KM12 in comparison with corresponding *Empty-*KM12 and *shCTL*-KM12 controls. Mean ± S.D., *n* = 3, **p < 0.01, *p < 0.05, two-tailed Student’s *t*-test. **e**, **f** Images and statistics of tumors isolated from subcutaneous xenografts in nude mice injected with *TRIM65-*KM12 cells and *Empty-*KM12 control. ns not significant (*p* > 0.05), two-tailed Student’s *t*-test. **g**, **h** Statistics and representative images of wound-healing assays of *TRIM65-*KM12 and *shTRIM65*-KM12 in comparison with the corresponding *Empty-*KM12 and *shCTL*-KM12 controls. Mean ± S.D., *n* = 3, *p < 0.05, ns not significant, two-tailed Student’s *t*-test. **i**, **j** Statistics and representative images of migration assays performed with *TRIM65-*KM12 and *shTRIM65*-KM12 to show the ability of TRIM65 to regulate cell migration, in comparison with the corresponding *Empty-*KM12 and *shCTL*-KM12 controls. Mean ± S.D.; *n* = 3, **p < 0.01, *p < 0.05, two-tailed Student’s *t*-test. (**k**, **l**) Statistics and representative images of invasion assays performed with *TRIM65-*KM12 and *shTRIM65*-KM12 to show the ability of TRIM65 to regulate CRC cell invasion, in comparison with corresponding *Empty-*KM12 and *shCTL*-KM12 controls. Mean ± S.D.; *n* = 3, **p < 0.01, *p < 0.05, two-tailed Student’s *t*-test

We next investigated whether TRIM65 is involved in the malignant progression of CRC. The efficiency of overexpression and knockdown of TRIM65 was confirmed (Figure S1a and S1b). TRIM65 overexpression promoted cell proliferation compared with that of the control (Figs. 2a and S1C); on the other hand, interference of TRIM65 expression by shRNA inhibited cell proliferation (Figs. 2b and S1d). The effect of TRIM65 expression on cell proliferation was further confirmed by colony formation assays (Fig. 2c, d). We also tested whether TRIM65 expression in cancer cells affects tumor growth in vivo. Xenograft tumors were established and measured according to previously reported procedures [18]. Tumor weights in the TRIM65-KM12 group were significantly heavier than those in the control group (Fig. 2e, f), and tumors in the shTRIM65-KM12 group were lighter (Fig. S1e and f).

We next tested whether the TRIM65 expression level is related to cancer cell migration and invasion behavior. Stable overexpression of TRIM65 (*TRIM65-*KM12) enhanced cell movement, resulting in higher migration distance (Fig. 2g, not statistically significant) and greater numbers of migrating cells (Fig. 2i); TRIM65 knockdown (*shTRIM65-*KM12) remarkably inhibited cell migration (Fig. 2h, j). To further assess the impact of TRIM65 on cell invasion, Transwell filters were precoated with Matrigel. We found an increasing number of *TRIM65-*KM12 cells invaded, and the cell invasion ability was inhibited in *shTRIM65*-KM12 cells (Fig. 2k, l). Collectively, these findings show that TRIM65 promotes cell growth, migration, and invasion in vitro, suggesting that TRIM65 also exerts oncogenic effects in CRC.

### TRIM65 modulates Rho GAP via ubiquitination of ARHGAP35

TRIM65 is an E3 ubiquitin ligase; therefore, by using iTRAQ, we identified potential ubiquitin-mediated downstream substrates (protein list in Supplemental file 2). Noticeably, GTPase-related genes are significantly enriched in the differential gene list, which includes seven members of the ARHGAP genes (Fig. S2b and Supplemental file 2).

**Fig. 3:**
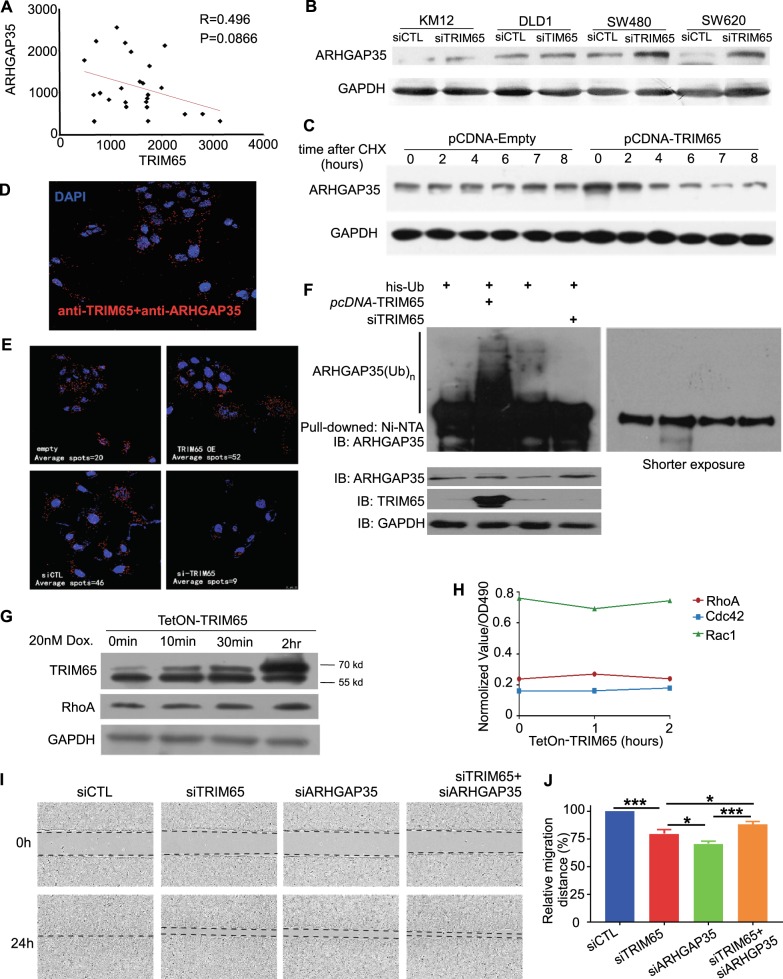
TRIM65 modulates Rho activity via ubiquitination of ARHGAP35. **a** Reverse correlation between protein expression levels of TRIM65 and ARHGAP35 in CRC tissues of patients. Each dot indicates relative protein expression level. **b** Representative immunoblots for ARHGAP35 expression in KM12 cells transiently transfected with siTRIM65 or siCTL. **c** Representative immunoblots indicate protein expression in KM12 cells with ectopic expression of TRIM65. Cycloheximide (CHX) (100 µg/ml) was used at 0 time point. **d** PLA on KM12 cells showing the interaction between TRIM65 and ARHGAP35 proteins. The red spots represent positive PLA signals, and the number of PLA signals represent TRIM65 and ARHGAP35 interaction spots in KM12 cells. **e** PLA with or without TRIM65 interference. PLA spots were counted and normalized to DAPI staining by a software accompanied with the assay kit (Trial version). The number of PLA signals per cell was increased in KM12 cells transiently transfected with *pCDNA-TRIM65* and reduced in KM12 cells transiently transfected with *siTRIM65* in comparison with the corresponding empty or control groups. **f** Representative immunoblots of an in vivo ubiquitination (Ub) assay of KM12 cells transiently cotransfected with *pCDNA-his-ubb* and *pCDNA-TRIM65* expression vectors or *siTRIM65* as well as their relative controls. Shorter exposure of the immunoblot is provided on the right panel. **g** Immunoblot analysis of time-dependent changes in protein levels of *TetOn-TRIM65* KM12 cells following doxycycline (20 µM) treatment. **h** G-LISA analysis of time-dependent changes in GTPase activity (normalized) of *TetOn-TRIM65-* KM12 cells following doxycycline treatment. **i** Representative images and (**j**) statistical analysis of wound-healing assays. Mean ± S.D., *n* = 3, ***p < 0.001, **p < 0.01, *p < 0.05, two-tailed Student’s *t*-test

Within the seven ARHGAP genes, ARHGAP35 (also known as P190RhoGAP) repeatedly appears in multiple enriched biological pathways. Therefore, we focused on TRIM65–ARHGAP35 regulation. We first determined the correlation of the protein level between TRIM65 and ARHGAP35 in CRC samples. There was a moderate correlation between these two proteins (*R* = 0.496, Fig. 3a). We also tested the correlation in CRC cell lines and observed similar results (Fig. S2c). We confirmed that the protein level of ARHGAP35 was influenced by the ectopic expression of TRIM65 in multiple cell lines, in which the protein level of ARHGAP35 was significantly accumulated when TRIM65 was knocked down by siRNA (Fig. 3b), while the mRNA level of ARHGAP35 was slightly increased in the same assay (Fig. S2d). We further confirmed this finding by treating cells with the protein synthesis inhibitor cycloheximide (CHX) to suppress de novo protein synthesis and observed that the degradation rate of ARHGAP35 was enhanced by TRIM65 expression (Fig. 3c).

Immunoprecipitation (IP) did not succeed in revealing a physical interaction between TRIM65 and ARHGAP35 (data not shown). We performed a PLA with TRIM65 and ARHGAP35 antibodies and found numerous spots in KM12 cells, in which each spot indicated one interaction loci in principle (Fig. 3d). The number of spots increased when TRIM65 was overexpressed and decreased after TRIM65 was knocked down by siRNA (Fig. 3e). These results suggested that TRIM65 directly bound to ARHGAP35 in the cells. In addition, we tend to think that TRIM65 ubiquitously localizes both in the nucleus and cytoplasm (Fig. S3).

Furthermore, a His-Ub plasmid was transfected into cells with either TRIM65 overexpression plasmid or *si* reagent. TRIM65 overexpression increased the ubiquitination level of ARHGAP35, while TRIM65 knockdown decreased the ubiquitination of ARHGAP35 (Fig. 3f). To test whether the TRIM65–ARHGAP35 interaction influences Rho GTPase activity, TRIM65 expression was induced by the Tet-on system, and protein level of RhoA did not change significantly when TRIM65 was induced (Fig. 3g). When TRIM65 was induced, the activity of RhoA increased after 1 h and then dropped back to the initial level after 2 h; the activity of Rac1 decreased after 1 h and then climbed back to the initial level after 2 h (Fig. 3h). Last, knockdown of TRIM65 and ARHGAP35 simultaneously restored the migration of KM12 cells compared with knockdown of TRIM65 only (Fig. 3i, j). Taken together, these results indicate that TRIM65 is involved in the modulation of Rho GAP via ubiquitin-mediated degradation of ARHGAP35.

### TRIM65 enhances cell migration through actin and microtubule cytoskeleton remodeling

**Fig. 4:**
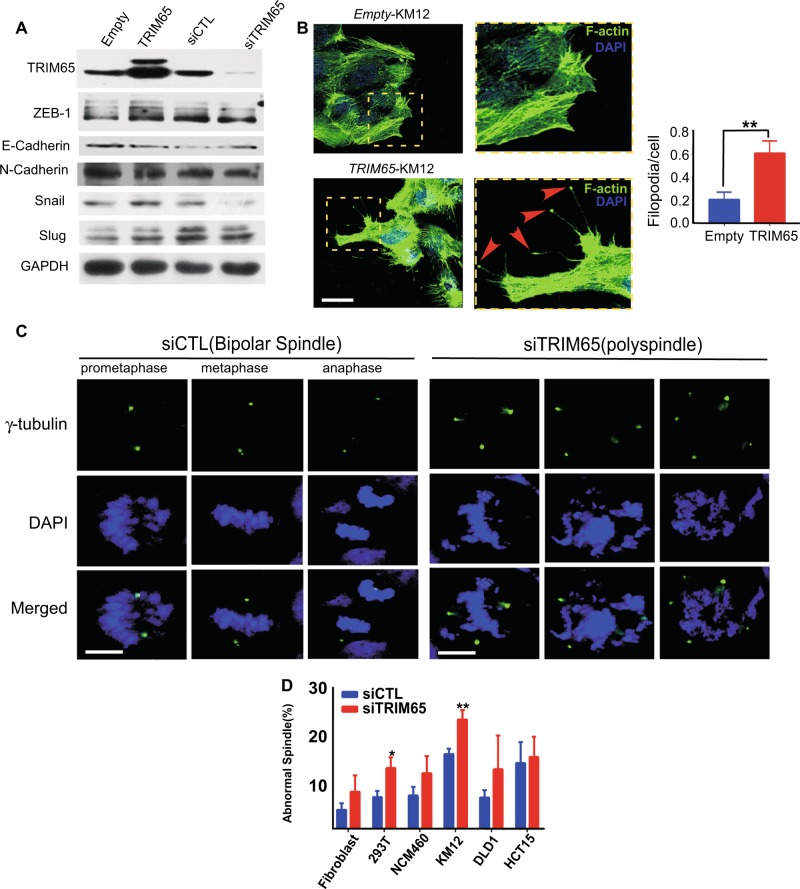
TRIM65 enhances cell migration through actin and microtubule cytoskeleton remodeling. **a** Representative immunoblots of EMT markers. **b** Images and statistics of the number of filopodia and focal adhesion of KM12 cells that were stably transfected with *TRIM65* or its control *Empty*, by using Alexa Fluor 488 phalloidin staining for the actin cytoskeletal fibers observed by fluorescence confocal microscopy. Filopodia/focal adhesion are indicated by red arrow heads in dash yellow box. At least 100 cells for each group were counted. Mean ± S.D, *n* = 3, **p < 0.01, two-tailed Student’s *t*-test. Scale bar: 100 µm. **c** Representative images of the mitotic spindle apparatus analyzed by immunofluorescence microscopy after cells were stained for γ-tubulin (green) and DAPI (blue). Left panel is siCTL with normal spindle and right panel is abnormal spindle (polyspindle) after siTRIM65. Scale bar: 12 μm. **d** Histogram shows the percentage of abnormal spindles in mitotic cells per experiment (siTRIM65 compared with siCTL, at least 100 cells counted for each cell line). Mean ± S.D.; *n* = 3, *p < 0.05, **p < 0.01, two-tailed Student’s *t*-test

Epithelial-to-mesenchymal transition (EMT) is considered one of the major characteristics when cells initiate metastatic programs in many cancer types. However, western blotting showed that the protein levels of several prototypic EMT markers barely or minimally changed when TRIM65 was overexpressed or knocked down by transient transfections, respectively (Fig. 4a). Therefore, we did not investigate the relationship between TRIM65 expression and EMT.

We established that ARHGAP35 is a substrate for TRIM65. ARHGAP35 is a known inhibitor of Rho GTPase, and RhoA has established functions in actin cytoskeleton remodeling. We observed rich filament-like protrusions extended from the leading edge of migratory cells with TRIM65 stably overexpressed (differential interference contrast microscopy, Fig. S2a). These protrusions were actin-rich filaments, as shown by phalloidin staining (Fig. 4b, left panel). Transient transfection of *pcDNA-TRIM65* reveal similar phenotype (Fig. S2e). The filaments appeared to be filopodia, which are actin-rich finger-like protrusions that extend from the plasma membrane. However, some of the filaments had a tip more swollen than the filament body itself, whose shape suggested a focal adhesion (arrow head, Fig. 4b). We found that exogenous TRIM65 induced more focal adhesion in KM12 cells than the empty control, which was confirmed by counting the number of focal adhesion and averaging it to the DAPI spots (right panel, Fig. 4b).

ARHGAP35 was also reported to be involved in spindle function. When TRIM65 was knocked down in CRC KM12 cells, a significantly increasing number of abnormal spindles (more than two spindle pole bodies, as indicated by γ-tubulin staining) was found in comparison with the *si* control (Fig. 4c). We also found a similar trend in several other cell types, including normal and cancer cell lines (Fig. 4d). And when TRIM65 was knocked down in fibroblast cells, no significantly higher percentage of abnormal spindles was observed (Fig. 4d). These results suggest that TRIM65 might be more strictly required for cancer cell division.

### The oncogenic activity of TRIM65 is regulated by phosphorylation

**Fig. 5:**
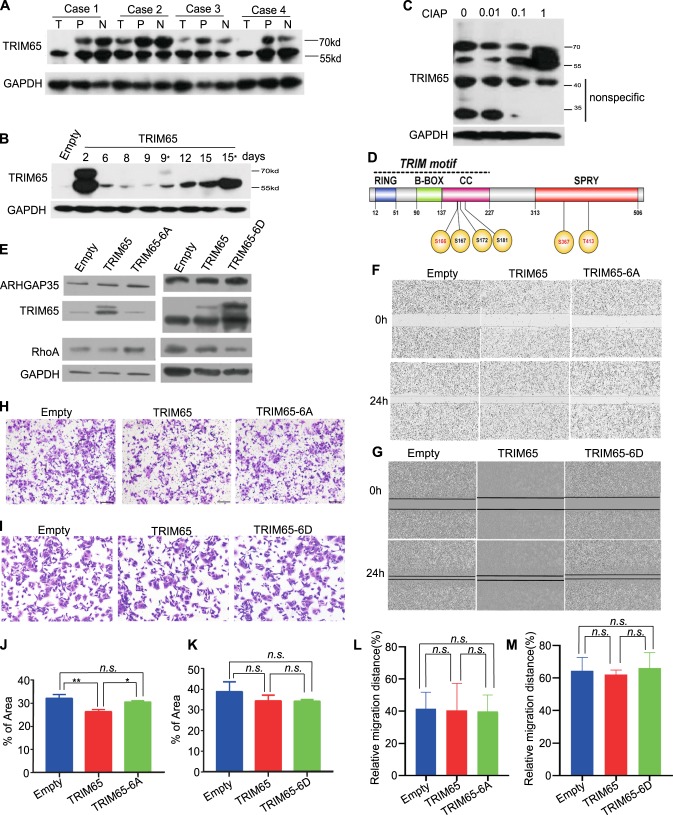
The oncogenic activity of TRIM65 is regulated by phosphorylation. Representative immunoblots of TRIM65 in CRC tissue, matched peritumoral, and normal tissue. T tumor tissue, P peritumor tissue (~2 cm away from tumor), N normal colon or rectal tissue (~5 cm away from the tumor). **a** Immunoblots of the protein expression of TRIM65 in KM12 cells at different days after transient or stable transfection (days 9 and 15 indicate with puromycin selection) with the TRIM65 overexpression vector. **b** Representative immunoblots of TRIM65 in KM12 cells treated with CIAP. The TRIM65 isoform (~68 kd) was partially transformed to 57-kd protein when KM12 cells were treated with CIAP phosphatase. **c** Schematic of the domain structure of TRIM65. TRIM65, consisting of a truly interesting new gene (RING) domain, a B-box domain, and a coiled-coil domain (CC), is followed by distinct C-terminal SPIa and the ryanodine receptor (SPRY) domain; phosphorylation residues S167, S367, and T413 are unambiguously identified, while S166, S172, and S181 are possible ones according to MS/MS peptide mapping. **d** Analysis of protein expression levels in KM12 cells with transiently ectopic expression of *TRIM65* (*pLenti-TRIM65*) or *TRIM65-6A* (*pCDNA-TRIM65-6A*) or *TRIM65-*6D (*pCDNA-TRIM65-6D*) compared with the empty control. **f** Representative images and (**l**) statistical analysis of wound-healing assays of KM12 cells with transiently ectopic expression of *TRIM65* (*pLenti-TRIM65*) or *TRIM65-6A* (*pCDNA-TRIM65-6A*) compared with the empty control. Mean ± S.D., *n* = 3, ns not significant (*p* > 0.05), two-tailed Student’s *t*-test. **g** Representative images and (**m**) statistical analysis a wound-healing assays of KM12 cells with transiently ectopic expression of *TRIM65* (*pLenti-TRIM65*) or *TRIM65-6D* (*pCDNA-TRIM65-6D*) compared with the empty control. Mean ± S.D., *n* = 3, ns not significant (*p* > 0.05), two-tailed Student’s *t*-test. **h** Representative images and (**j**) statistical analysis of invasion assays of KM12 cells with transiently ectopic expression of *TRIM65* (*pLenti-TRIM65*) or *TRIM65-6A* (*pCDNA-TRIM65-6A*) compared with the empty control. Mean ± S.D., *n* = 3, **p < 0.01, *p < 0.05, ns not significant (*p* > 0.05), two-tailed Student’s *t*-test. **i** Representative images and (**k**) statistical analysis of invasion assays of KM12 cells with transiently ectopic expression of *TRIM65* (*pLenti-TRIM65*) or *TRIM65-6D* (*pCDNA-TRIM65-6D*) compared with the empty control. For **j**, **k**, **l**, and **m**, mean ± S.D., *n* = 3, ns not significant, two-tailed Student’s *t*-test

Previously, TRIM65 was reported to be phosphorylated. We noticed that there were two discrete bands when performing immunoblotting for TRIM65: a 57- and a 68-kd band. The 57-kd band was the main band according to the product manual. Interestingly, the 68-kd band appeared to fade or disappear in tumor samples compared with that in matched peritumor and normal tissues (Fig. 5a). When the TRIM65 overexpression plasmid with the puromycin selection marker was transfected into the CRC cell line, the 68-kd band eventually disappeared either with or without puromycin selection (Fig. 5b: days 9 and 15, with stars indicating puromycin selection). The 68-kd band is sensitive to calf intestinal alkaline phosphatase (CIAP) treatment (Fig. 5c). Therefore, we believe that TRIM65 might also be translationally modified by phosphorylation in CRC.

To this purpose, we identified phosphorylation residues from the mass spectrometry (MS) analysis of TRIM65 IP: three sites (S167, S367, and T413) confirmed and three sites highly possible (S166, S172, and S181) according to the MS/MS maps (Supplemental file 3). Within the six residues, four residues are in the N-terminal coil-coil domain, and two residues are in the C-terminal SPRY domain (illustrated in Fig. 5d). We constructed a phospho-inhibiting mutation for TRIM65 with all six phosphorylation residues mutated to alanine (S/T to A) and a phospho-mimicking mutation with all six residues mutated to aspartic acid (S/T to D). Subsequent transfection of the plasmid into the CRC cell line showed that the 68-kd band disappeared in the TRIM65-6A transfection, while it appeared to be more apparent in the TRIM65-6D transfection, when compared with the 57-kd band (Fig. 5e). Interestingly, while the protein level of ARHGAP35 was not sensitive to the mutational state of TRIM65, the protein level of RhoA seemed to be influenced by the mutational state of TRIM65 (Fig. 5e). Phenotypically, both wild-type TRIM65 and TRIM65-6A/6D transient transfections did not show a significant change of cell migration ability (Fig. 5f, l; Fig. 5g, m), but the invasion ability was higher in the TRIM65-6A transient transfection compared with the wild-type TRIM65 transfection (Fig. 5h, j). The invasion ability of the TRIM65-6D transient transfection was comparable with that of the wild-type TRIM65 transfection (Fig. 5i, k). In sum, TRIM65 was also translationally modified by phosphorylation in CRC, and the phosphorylation state of TRIM65 seemed to attenuate its oncogenic activity.

### TRIM65 promotes tumor cell metastasis in vivo

**Fig. 6:**
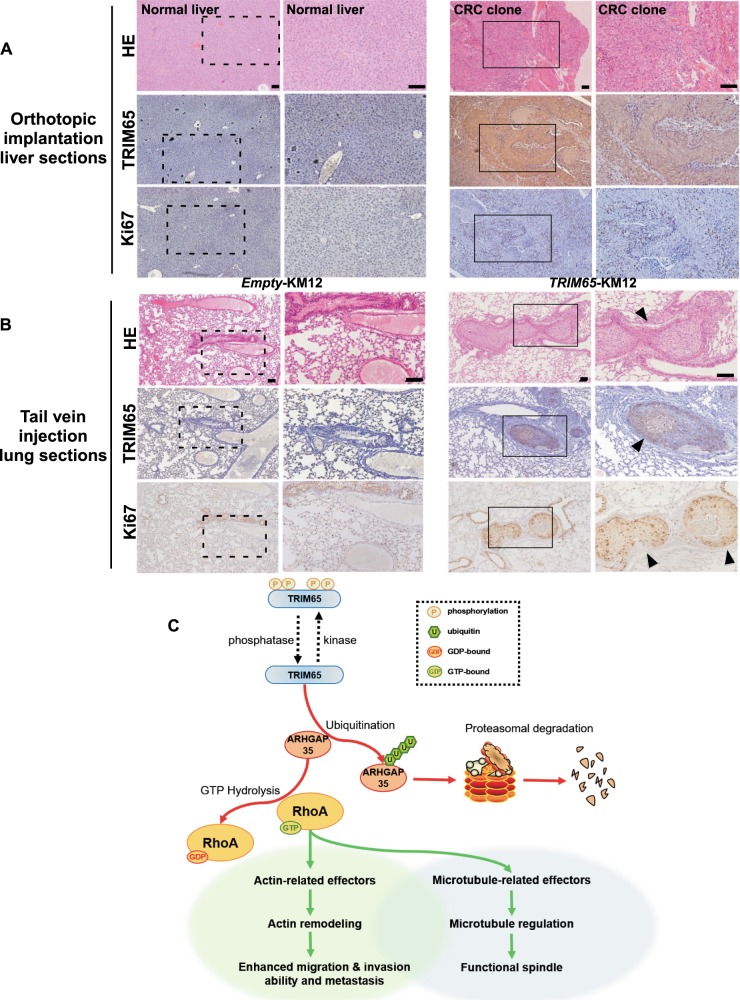
TRIM65 promotes tumor cell metastasis in vivo. **a** Representative images of H&E and IHC (TRIM65 and Ki67)-stained tumors and liver sections of nude mice with the orthotopic implantation of tumor clumps formed with *TRIM65-*KM12 or *Empty-*KM12 cells. Scale bar: 100 µm. **b** Representative H&E and IHC (TRIM65 and Ki67) staining images of lungs with or without metastasis of CRC clones of nude mice in nude mice intravenously injected with *TRIM65-*KM12 or *Empty-*KM12 cells. Scale bar: 100 µm. **c** Integrative model illustrating the role of TRIM65 in cytoskeleton regulation

Clinical parameter analysis and in vitro cell-based assays indicated that TRIM65 promotes invasion and migration of CRC cells, thereby facilitating the metastasis of cancer cells in vivo. Thus, we tested the prometastatic functions of TRIM65 in vivo using the orthotopic implantation of cancer cells to construct a nude mouse metastatic model. Microscopic examination of liver sliced sections revealed metastatic subclones in mouse livers in the *TRIM65*-KM12 group (one out of four mice, solid box, right panel, Fig. 6a), while no liver metastasis was found in the control group (left panel, Fig. 6a). IHC staining revealed that TRIM65 and Ki67 were positively stained in the liver metastasis tumor clone (middle and low panels, Fig. 6a).

For a more direct assessment of metastatic potential, mice were intravenously injected with TRIM65-KM12 or Empty-KM12 cells, then both livers and lungs sliced sections were microscopically examined for the presence of metastatic tumors. We found that *TRIM65*-KM12 cells resulted in lung metastasis formation in vivo (solid box, right panel, Fig. 6b), while no metastatic tumor was seen by microscopic examination in the *Empty*-KM12 group (left panel, Fig. 6b). Among all 15 mice injected with *TRIM65*-KM12 cells, four mice had metastatic cancer (with 1, 3, 4, and 6 nodules, respectively). IHC was performed to confirm the high expression of TRIM65 and to indicate proliferating cells by Ki67 staining (middle and low panels, Fig. 6b). Taken together, these data indicate that TRIM65 could promote the invasive and metastatic capacity of colorectal cells in vivo.

## Discussion

In this study, we found that TRIM65 is a prognosis-associated marker that is dysregulated in CRC. Both the transcription and protein levels of TRIM65 are commonly elevated in CRC and are associated with poorer overall survival. Patients with relapse and metastasis had an even higher level of TRIM65. In vitro and in vivo assays demonstrated the requirement of TRIM65 for CRC cell proliferation, invasion, and migration. TRIM65 also promoted CRC metastasis in a mouse model. We identified a novel TRIM65 downstream substrate, ARHGAP35, which is a GAP with established function in RhoA inactivation [19–21]. RhoA is generally overexpressed in cancer and is a key GTPase required for cell migration by reorganization of the actin cytoskeleton in temporal and spatial fashion [20, 22]. TRIM65 binds to ARHGAP35 and enhances its ubiquitination, targeting ARHGAP35 for proteasomal degradation. In summary, we propose a model of TRIM65 in CRC metastasis through the TRIM65/ARHGAP35/RhoA axis (Fig. 6c).

Amino acid substitutions of the identified phosphorylation sites in TRIM65 (S–A, mimicking dephosphorylation status) did not abolish the migration ability of cancer cells, suggesting that the dephosphorylated form of TRIM65 protein might be more related to TRIM65 function in tumor progression. It is worth mentioning that when we conducted most of the cell-based assays, we generated both transient-transfection and stable-transfection cell lines and compared them in parallel. Some of the phenotypes, such as cell proliferation, migration, and invasion ability, were prominent only in the stably transfection cell lines. Transient transfection yields a pool of phosphorylated and unphosphorylated forms of TRIM65 protein, thereby attenuating the cells in terms of aggressiveness. Phospho-TRIM65 might be counter selected when the cells are passaged. Efforts to introduce phospho-mutations in TRIM65 complete knockout background, and to identify the corresponding protein kinase and phosphatase should be made to unravel the regulatory mechanisms and the biological significance of TRIM65 phosphorylation.

To this end, it is tempting to conclude that TRIM65 is an important hub for signaling cross talk in general, which also implies that the functions of TRIM65 in a physiological or disease context should be considered. For example, in a study by Yang et al. [16], HMGA1 seems to be an upstream transcription factor (TF) for TRIM65 regulation. We looked for a correlation between HMGA1 and TRIM65 in both the TCGA CRC data set and our cohort, and failed to see evidence for that finding. We also tested several typical transcription factors (TFs), such as MYC and C-JUN, and did not observe a significant change in TRIM65 transcription when these TFs were knocked down. Therefore, it is necessary to search for the upstream TFs that modulate the transcription of TRIM65. Since there are many predicted TF binding sites in the gene promoter of TRIM65, multiple TFs might be cooperating to regulate TRIM65. In addition, we did not detect significant protein-level change of β-catenin when TRIM65 is either overexpressed or knocked down (data not shown).

It is worth to mention that few studies reported the localization and functionality of TRIM65 based on relatively large epitope tag like GFP in frame-fused expression. It appeared that while no epitope tag or small tag like FLAG showed ubiquitous signal across the cell when performing IF with TRIM65 antibody (Fig. S3a and b), *GFP-TRIM65* fusion expression showed punctate in some cells when performing IF with GFP antibody (Fig. S3c). Therefore, caution should be payed when choosing expression plasmid to investigate TRIM65 regulation.

Even though the EMT is not always required for cancer metastasis, e.g., lung cancer [23] and pancreatic cancer [24], it is still commonly seemed in many other cancer types, including CRC. Our data suggests that TRIM65 is unlikely to be upstream of EMT programme. Ectopic forced expression of TRIM65 in the cell resulted in increases in migration-related structures and filopodia/focal adhesion. These structures are critical for efficient cell movement and migration. More often, two alternate forms of actin machinery (filopodia and lamellipodia) coexist at the leading edge of most motile cells. Both filopodia/lamellipodia and focal adhesion have been implicated in cell migration and invasion, as well as chemosensing both in vitro and in vivo [25]; therefore, we concluded that TRIM65 enhances cancer cell migration by modulating the actin cytoskeleton to produce more filopodia/lamellipodia and focal adhesions. Similar actin-rich architecture had been reported to enhance metastasis in CRC peritoneal metastasis model [26]. More filopodia/focal adhesions enhance cell motility and coping with the microenvironment along the metastasis route, thereby increasing the likelihood of ultimately successful metastasis. Noticeably, TRIM65 is also required for cell division, as knockdown of TRIM65 impairs spindle function. Detailed dissection of TRIM65 in cytoskeleton regulation is required to pinpoint the underlying specific mechanisms. Because overexpression of TRIM65 is significantly associated with metastatic potential, TRIM65 may serve as a valuable prognostic factor to predict metastasis in patients. Blocking the interaction between fascin and actin by small molecules has been showing great promise to stop cancer metastasis and relapse [6, 27]. Given that TRIM65 also participates in spindle function, the TRIM65–ARHGAP35 interaction might be a candidate therapeutic target.

## Materials and methods

### Patients and tissue samples

For mRNA quantification, we obtained frozen tissue paired samples stocked in RNAlater solution (Invitrogen, Thermo Fisher Scientific, USA) from 194 CRC patients with primary CRC tissues and adjacent normal tissues (160 samples had normal tissues) who had undergone surgery between October 2010 and July 2016 at the Sixth Affiliated Hospital of Sun Yat-sen University (SYSU). Of the 194 patients, 102 had disease relapse or metastasis within 3 years after surgery. The adjacent normal tissues were derived from ~5 cm away from the tumor border.

We also constructed tissue microarrays of primary CRC from 374 patients from the Sixth Affiliated Hospital of SYSU. None of the patients included received adjuvant radiotherapy or chemotherapy. We excluded the sample when that core on the microarray was missing during the experimentation.

### Cell culture and transfection

HCT8, KM12, Caco-2, DLD-1, HCT116, LoVo, HT-29, SW480, SW620, RKO, and HCT15 CRC cell lines; NCM460 cells; fibroblasts; and 293 cells with SV40-T antigen (293 T) were used. Cells were obtained from ATCC. siRNAs used in this study are as follows: siTRIM65: 5′ -AGC CAA GCC UGU GGA CUU A-3′ (multiple siRNA were tested). Nontargeting negative control siRNA (*siCTL*) was commercially synthesized (RiboBio Co., Ltd., China). KM12 and HCT8 were selected to generate overexpression/interference stable cell lines, termed *TRIM65-KM12/-HCT8* and *shTRIM65-KM12/-HCT8*, with controls termed *Empty-KM12/-HCT8* and *shCTL-KM12/-HCT8*, respectively. Other plasmids are listed in Supplemental file 1.

### qRT-PCR analysis

The sequences of the PCR primers were as follows: TRIM65, forward: 5′-AAG CAG CCA GAT CCA GAA CTC-3′ and reverse: 5′-CTC AGT GCT GTC GTG TG-3′; β-actin, forward: 5′-TTG TTA CAG GAA GTC CCT TGC C-3′ and reverse: 5′-ATG CTA TCA CCT CCC CTG TGT G-3′; ARHGAP35, forward: 5′-AGA AAG AGC CGG TTG GTT CAT-3′ and reverse: 5′-AAC ATA GCC AAA GAG GCC TTA CG-3′. β-actin was used for normalization.

### Western blot

The primary antibodies used were as follows: TRIM65 (1:1000, HPA021578, Sigma-Aldrich), GAPDH (1:10000, 60004-1-Ig, Proteintech), and ARHGAP35 (GRLF1, 1:1000, 26789-1-AP, Proteintech). The secondary antibodies were anti-rabbit IgG-HRP and anti-mouse IgG-HRP (7074 S and 7076, Cell Signaling Technology). Other antibodies are listed in Supplemental file.

### HE and immunohistochemistry assay

IHC for TRIM65 (1:100, HPA021575, Sigma-Aldrich) was performed on CRC tissue microarrays. The scores were independently assessed by two technicians. The IHC score of 4 was selected as the cutoff value for defining high and low expression.

### Cell proliferation and colony formation assays

The real-time cell analyzer (RTCA, xCELLigence system, ACEA Biosciences, Inc.) was used for the cell proliferation assay. A total of 5000 cells were seeded into each well. The combination index value was recorded every 30 min automatically.

For colony formation assays, stable cells were constructed. Cells were collected and seeded into six-well plates at a density of 200 per well and then incubated at 37 °C for 6 days. Colonies were fixed with 4% paraformaldehyde, stained with 0.1% crystal violet, and counted.

### Wound-healing assay

Standard scratch-based assays were performed. Each assay was repeated three times.

### Migration and invasion assay

Boyden chambers (Corning, NY, USA) were used to perform the migration assay. For invasion assay, filters (8-μm pore size) were precoated with Matrigel (Corning, NY, USA), and experiments were repeated at least three times.

### Mouse experiments

Female BALB/c nude mice, 4–5 weeks old, were randomly divided into different experimental groups.

### Xenograft mouse model

Mice were injected subcutaneously with 5 × 10^6^ KM12 cells in a 200-µl volume into the left flanks. After 1 month, tumors were dissected and weighed. Mice were anesthetized using CO_2_ gas and euthanized by cervical dislocation.

### In vivo metastasis assay

Both orthotopic implantation and tail vein injection were done to access the metastasis potential of CRC cells.

### Immunofluorescence assay

Cells were fixed and stained according to the IHC antibody protocols, and slides were visualized by Leica TCS-SP8 confocal microscopy equipped with 10×, 20×, 40×, and 100× objectives (Mannheim, Baden-Wuerttemberg, Germany).

### iTRAQ

Two different plasmids (OE-TRIM65, OE-control) and two si reagents (siTRIM65, siControl) were transfected into KM12 cells in parallel and prepared for iTRAQ labeling and MS analysis. Enrichment analysis for differential proteins were done in FunRich_V3 [28].

### Coimmunoprecipitation, in-gel tryptic digestion, and mass spectrometry (MS)

Plasmid (pcDNA3-cFlag-TRIM65) and the empty control were transfected into cells to make overexpression of TRIM65 protein. Protein preparation and MS setup were following the protocol by Keck et al. [29], with minor modifications.

### Proximal ligation assay (PLA)

Interaction between TRIM65 and ARHGAP35 was detected by using an in situ PLA kit (Sigma-Aldrich, Darmstadt, Germany) in the KM12 cell line, according to the kit protocol.

### Ubiquitination assay

KM12 cells were transfected with the indicated *pCDNA-TRIM65* and *pCDNA-Empty* plasmids or TRIM65 siRNA (termed *siTRIM65)* with scramble control (*siCTL*). The ubiquitinated proteins were pulled down with Ni-NTA magnetic agarose (#78605, Thermo Fisher Scientific, USA) following the manufacturer’s instructions. The protein complexes were then probed with anti-ARHGAP35 antibody to visualize the level of ubiquitination.

### Turnover assay

Cells were transfected with *pCDNA-TRIM65* and *pCDNA-Empty* plasmids and incubated in 5% (v/v) CO_2_ at 37 °C for 24 h. Cycloheximide (CHX, MedChem Express) was then added to the media at a final concentration of 100 μg/ml for the indicated times. The cells were harvested, and ARHGAP35 protein levels were analyzed by immunoblotting.

### G-LISA assay

RhoA, Rac1, and Cdc42 were quantified using the G-LISA Rho Activation Assay Biochem Kit (Cytoskeleton, Inc.), following the manufacturer’s instructions.

### Statistical analysis

Chi-square tests and one-way ANOVA were used to assess differences in clinical variables between the CRC cohorts. Kaplan–Meier survival analyses were used to compare survival times among CRC patients based on TRIM65 expression; the log-rank test was used to generate *p* values. Cox proportional hazards regression analyses were used to assess the effect of clinical variables on patient survival. Univariate and multivariate analyses were used to assess the influence of clinical variables on survival. The *p* values and hazard ratios are indicated. Differences between groups were evaluated using a two-tailed Student’s *t*-test or a Mann–Whitney *U-*test. All statistical analyses were performed using SPSS 22.0 or GraphPad Prism 7. Paired samples were compared using a paired-sample *t*-test.

### Study approval

A written informed consent for each patient about tissue sampling was obtained and the study was reviewed and approved by the Medical Ethics Committee of the Sixth Affiliated Hospital, Sun Yat-sen University. The animal studies were reviewed and approved by the Animal Care and Use Committee of SYSU.

## Supplementary information

S1

S2

S3

Dataset 1

supplementary material file

Dataset 2
