# Supplementary figures and images for: Ubiquitin ligase TRIM65 promotes colorectal cancer metastasis by targeting ARHGAP35 for protein degradation
Source: Oncogene. 2019 Jul 22;38(37):6429–44. doi: 10.1038/s41388-019-0891-6 (PMC6756236; doi:10.1038/s41388-019-0891-6)

**Figure S1****A**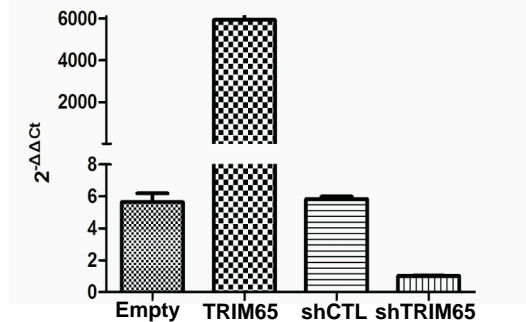**B**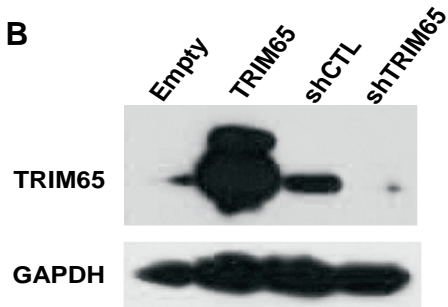**C**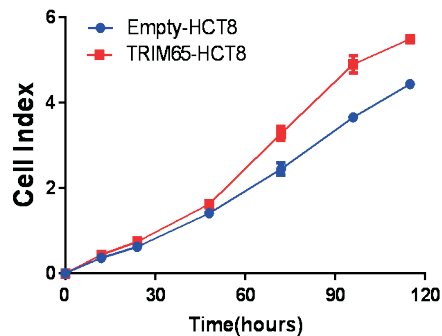**D**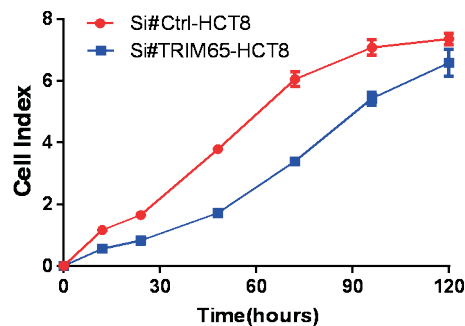**E**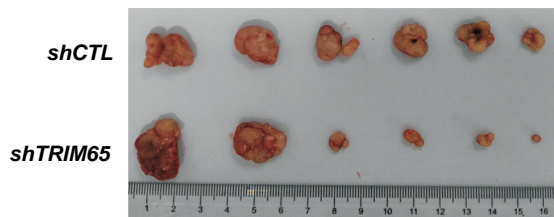**F**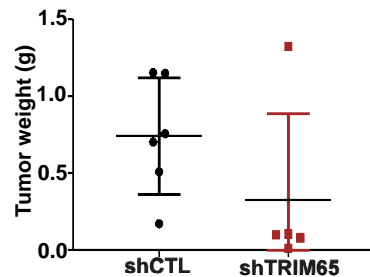

Supplement: Supplementary file 1 — S1 [file 41388_2019_891_MOESM1_ESM.pdf]

**Figure S2****A**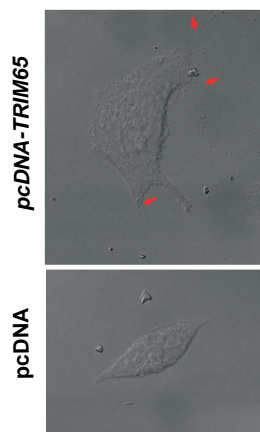**B**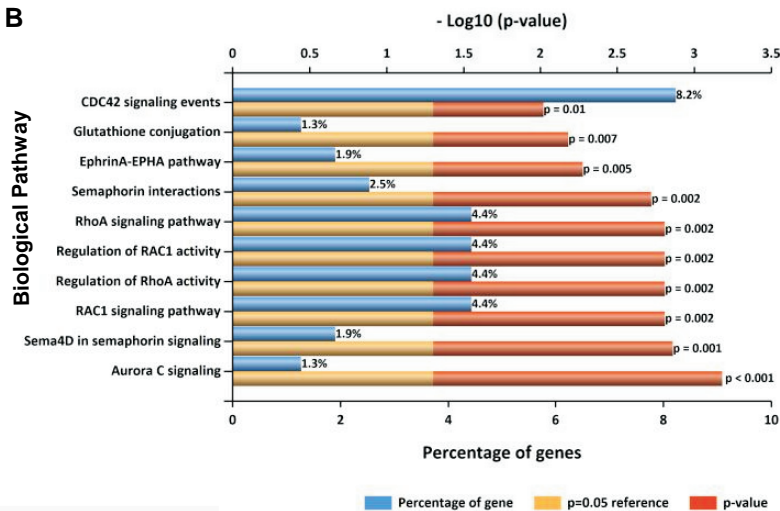**C**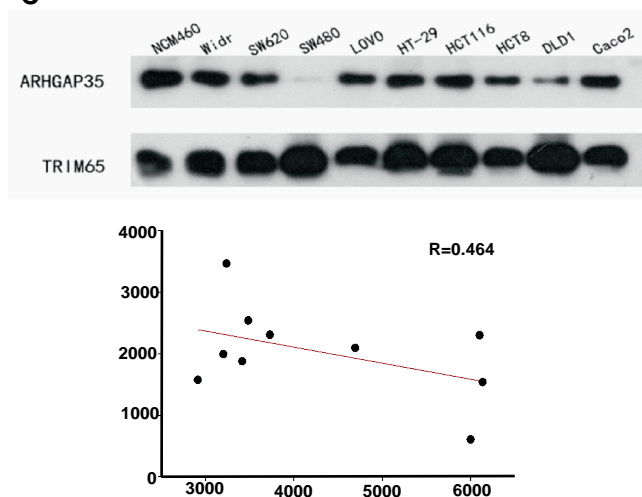**D**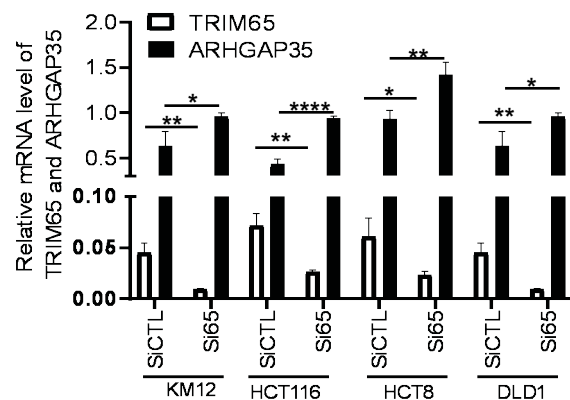**E**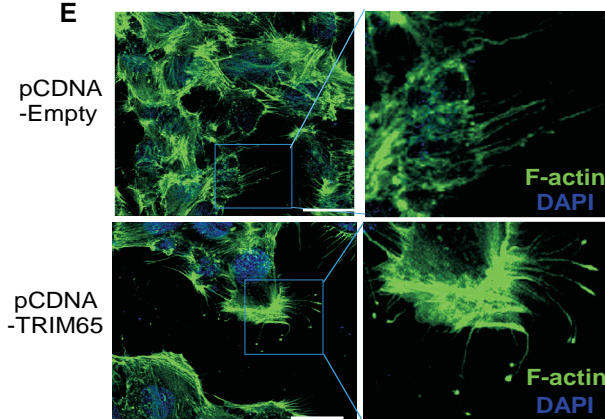

Supplement: Supplementary file 2 — S2 [file 41388_2019_891_MOESM2_ESM.pdf]

**Figure S3**

**A**

*pCDNA-Empty* KM12

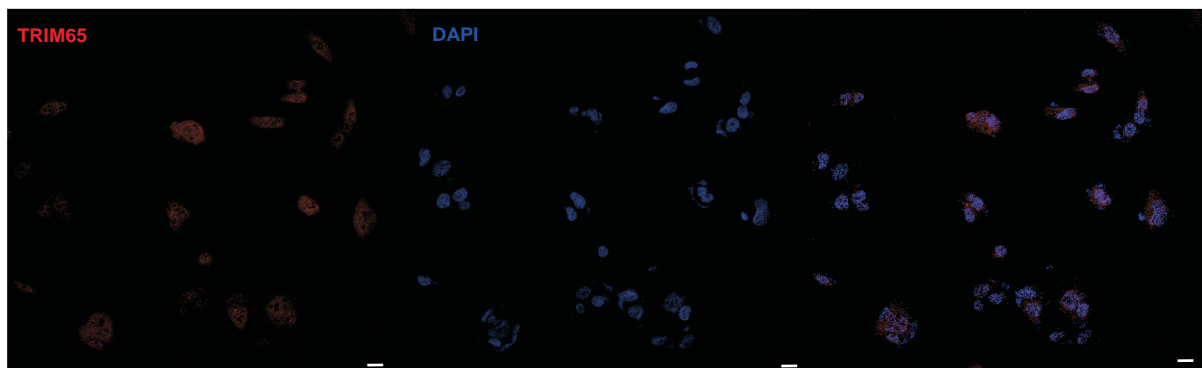

**B**

*pCDNA-TRIM65* KM12

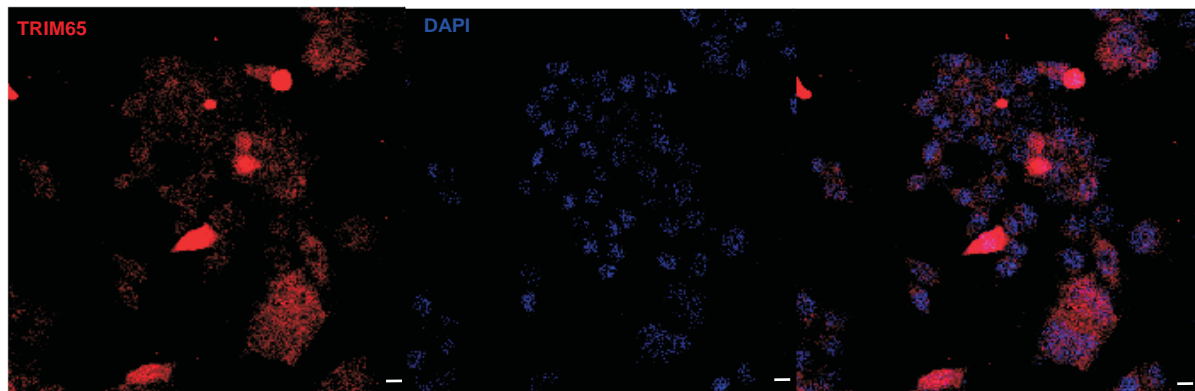

**C**

*pEGFP-N1-TRIM65* KM12

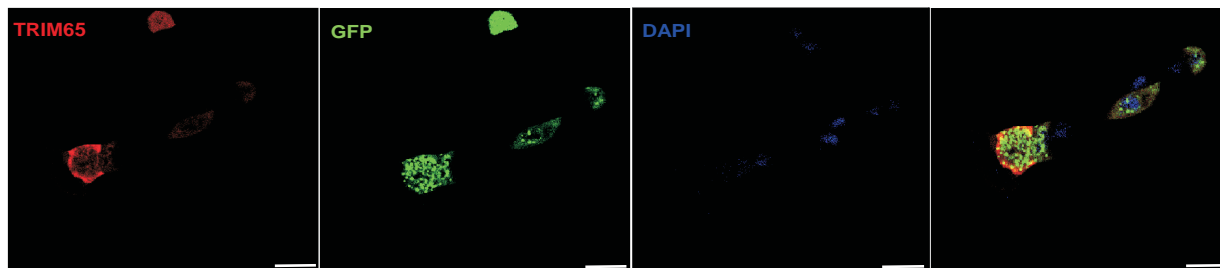

Supplement: Supplementary file 3 — S3 [file 41388_2019_891_MOESM3_ESM.pdf]
