# Supplementary material for: Ubiquitin ligase TRIM65 promotes colorectal cancer metastasis by targeting ARHGAP35 for protein degradation
Source: Oncogene. 2019 Jul 22;38(37):6429–44. doi: 10.1038/s41388-019-0891-6 (PMC6756236; doi:10.1038/s41388-019-0891-6)
Supplement: Supplementary file 5 — supplementary material file [file 41388_2019_891_MOESM5_ESM.docx]

**MS mapping for phospho-peptides of TRIM65**

**Supplemental Materials and Methods**

***Patients and Tissue Samples***

For mRNA quantification, we obtained frozen tissue paired samples stocked in RNAlater solution (Invitrogen, Thermo Fisher Scientific, USA) from 194 CRC patients with primary CRC tissues and adjacent normal tissues (160 samples had normal tissues) who had undergone surgery between October 2010 and July 2016 at the Sixth Affiliated Hospital of Sun Yat-sen University (SYSU). Of the 194 patients, 102 had disease relapse or metastasis within 3 years after surgery. The adjacent normal tissues were derived from approximately 5 cm away from the tumor border.

We also constructed tissue microarrays of primary CRC from 374 patients from the Sixth Affiliated Hospital of SYSU. None of the patients included received adjuvant radiotherapy or chemotherapy. We excluded the sample when that core on the microarray was missing during the experimentation.

***Cell Culture and Transfection***

HCT8, KM12, Caco-2, DLD-1, HCT116, LoVo, HT-29, SW480, SW620, RKO and HCT15 CRC cell lines; NCM460 cells; fibroblasts; and 293 cells with SV40-T antigen (293T) were used. Cells were obtained from ATCC or underwent authentication. siRNAs used in this study were as follows: siTRIM65-1: 5'-AGC CAA GCC UGU GGA CUU A-3‘, siTRIM65-2: 5'-GUA GGA CCC UGA CCC UGU G-3', siARHGAP35-3: 5′- AAG AUG CAC AUU GUG GAG CAG-3′. We tested all three chose the siTRIM65-1 for the study. Nontargeting negative control siRNA (*siCTL*) was commercially synthesized (RiboBio Co., Ltd., China). KM12 and HCT8 were selected to generate overexpression/interference stable cell lines, termed *TRIM65-KM12/-HCT8* and *shTRIM65-KM12/-HCT8*, with controls termed *Empty-KM12/-HCT8* and *shCTL-KM12/-HCT8*, respectively. The following *in vitro* assays were conducted with the above stably transfected cell lines. Other plasmids and culturing detail are listed in Supplemental file 1 and 3.

***qRT-PCR Analysis***

The sequences of the PCR primers were as follows: TRIM65, forward: 5′- AAG CAG CCA GAT CCA GAA CTC -3′ and reverse: 5′- CTC AGT GCT GTC GTG TG -3′; β-actin, forward: 5′- TTG TTA CAG GAA GTC CCT TGC C -3′ and reverse: 5′- ATG CTA TCA CCT CCC CTG TGT G -3′; ARHGAP35, forward: 5'-AGA AAG AGC CGG TTG GTT CAT-3' and reverse: 5′-AAC ATA GCC AAA GAG GCC TTA CG-3'. β-actin was used for normalization. please refer to Supplemental file 3 for more detail.

***Western Blot***

Immunoblots were performed with whole cell lysates or tissue extracts prepared using T-PER tissue protein extraction reagent (Thermo Fisher Scientific, USA) supplemented with protease and phosphatase inhibitors (Millipore, Merck KGaA, Darmstadt, Germany). Protein samples were loaded onto Tris-glycine gels and then transferred to a 0.45 µm PVDF membrane (Millipore, Merck KGaA, Darmstadt, Germany). After saturation in Tris-buffered saline buffer supplemented with 5% powdered skim milk, the membranes were incubated with antibodies overnight at 4℃. Next, the membranes were washed three times with TBST for at least 30 min and probed with HRP-linked secondary antibodies for 1.5 h at room temperature. Protein bands were visualized with a chemiluminescence detection kit (Thermo Fisher Scientific, Inc.). After three washes with TBST buffer, a semiquantified analysis was carried out using ImageJ software (National Institutes of Health, Bethesda, MD, USA). The primary antibodies used in the western blots were as follows: TRIM65 (1:1000, HPA021578, Sigma-Aldrich), GAPDH (1:10000, 60004-1-Ig, Proteintech), and ARHGAP35 (GRLF1, 1:1000, 26789-1-AP, Proteintech). The secondary antibodies were anti-rabbit IgG-HRP and anti-mouse IgG-HRP (7074S and 7076, Cell Signaling Technology). Other antibodies are listed in Sup_File 1.

***HE and Immunohistochemistry Assay***

Immunohistochemistry (IHC) for TRIM65 (1:100, HPA021575, Sigma-Aldrich) was performed on CRC tissue microarrays. The protocol was followed according to the manufacturer's instructions (ORIGENE, SP-9000). The sections were deparaffinized in xylene and then rehydrated using graded ethanol. The slides were then placed in 3% hydrogen peroxide to quench endogenous peroxidase and then processed for antigen retrieval by microwave heating for 10 min in 10 mM citrate buffer (pH 6.0). The primary antibody was then incubated at 4°C overnight. The next day, the primary antibody was rinsed with phosphate-buffered saline (PBS), and a biotin-labeled goat anti-mouse/rabbit IgG polymer was added. HRP-labeled streptavidin was then added after rinsing with PBS. 3, 3', 4, 4'-Biphenyltetramine tetrahydrochloride (DAB) was applied and resulted in a brown precipitate at the antigen site. The slides were counterstained with hematoxylin (Huntz Enterprises, Inc.), mounted in nonaqueous mounting medium, and coverslipped. Expression levels were scored as a proportion of the immunopositive staining area (0%, 0; 1–25%, 1;26–50%, 2; 51–75%, 3; 76%–100%, 4) multiplied by the intensity of staining (0, negative; 1, weak; 2, moderate; 3, intense). The scores were independently assessed by two pathologists. The IHC score was selected as the cut-off value for defining high and low expression.

***Cell Proliferation and Colony Formation Assays***

The real-time cell analyzer (RTCA, xCELLigence system, ACEA Biosciences, Inc.) was used for the cell proliferation assay. A total of 5000 cells were seeded into each well. The combination index (CI) value was recorded every 30 min automatically.

For colony formation assays, stable cells were constructed. Cells were collected and seeded into six-well plates at a density of 200 per well and then incubated at 37°C for 6 days. Colonies were fixed with 4% paraformaldehyde, stained with 0.1% crystal violet and counted.

***Wound-Healing Assay***

Cells were plated into 6-well plates and allowed to grow to confluence. The FBS was then disposed of, and wounds were introduced by scraping the confluent cell cultures with the tip of a 200-μl pipette. Floating cells were removed before complete medium was added. The wound-healing process was monitored under a microscope. Each assay was repeated 3 times.

***Migration and Invasion Assay***

For the migration and invasion assays, 24-well Boyden chambers (Corning, NY, USA) were used. Filters (8-μm pore size) were used for estimating cell migration, and filters precoated with Matrigel (Corning, NY, USA) were used for estimating cell invasion. Cells were placed into the upper chamber in 0.3 ml of serum-free RPMI-1640 (4×10^4^ cells per filter). RPMI-1640 supplemented with 10% FBS was placed in the lower chamber as a chemoattractant and incubated for 16 h. Cells were fixed in 4% paraformaldehyde for 10 min at room temperature, stained with crystal violet for 1 min, and photographed under microscopy. The purple area was estimated by ImageJ. Experiments were repeated at least three times.

***Mouse Experiments***

Female BALB/c nude mice, 4–5 weeks old, were randomly divided into experimental groups.

*Xenograft Mouse Model*

Mice were injected subcutaneously with 5×10^6^ KM12 cells in a 200 µl volume into the left flanks. After 1 month, tumors were dissected and weighed. Mice were anesthetized using CO_2_ gas and euthanized by cervical dislocation.

*In vivo* *Metastasis Assay*

For orthotopic implantation, *TRIM65-* or *Empty-*KM12 cells were mixed with Matrigel and injected into the right flank of immunodeficient nude mice. Fourteen days after engraftment, tumors were extracted and cut into similar clumps (1 mm × 1 mm). Tumor clumps were transplanted and fixed at the mesenteric region of the ileocecum (Treves region) of immune-deficient nude mice. Mice were euthanized 61 days after injection, livers and lungs were fixed and cut in a series of sections, and tumors were counted using a light microscope. The total number of metastatic nodules in the livers and lungs of each mouse was assessed, and the *p*-value of the difference was calculated using the Mann-Whitney U test.

For tail vein injection, 1× 10^6^ KM12 cells were injected into the tail vein of nude mice (15 mice for each group). Mice were euthanized 21 days after injection, lungs were fixed and cut into a series of sections, and tumors were counted using a light microscope. The total number of metastatic nodules in the lungs of each mouse was assessed, and the *p*-value of the difference was calculated using the Mann-Whitney U test.

***Immunofluorescence Assay***

KM12 cells were grown on glass coverslips and transfected with the indicated plasmids *(pCDNA-TRIM65* and *pCDNA-Empty or siTRIM65 and siCTL*) 48 h later. For staining of TRIM65 and actin cytoskeleton by phalloidine**,** cells were fixed with 4% paraformaldehyde for 20 min, permeabilized for 5 min with 1% Triton X-100, and blocked with 3% bovine serum albumin (BSA) for 1 h. Immunofluoresence staining was performed using anti-ΤΡΙΜ65 as a primary antibody, which was detected via goat anti-rabbit Alexa 488-conjugated secondary antibody or by adding Alexa Fluor 488-Phalloidin (KGMP0012, KeyGEN BioTECH, Beijing, China) according to the manufacturer’s instructions and incubated for 20 min at room temperature, followed by staining cell nuclei with 4',6-diamidino-2-phenylindole (DAPI) (Sigma-Aldrich, Merck KGaA, Darmstadt, Germany). For γ-tubulin staining, cells were fixed for 10 min with precooled 100% methanol at -20°C and blocked for 1 h with 5% BSA. Immunofluorescence staining was performed using anti-γ-tubulin (1:1000, T5192, Sigma-Aldrich) as the primary antibody, which was detected via goat anti-rabbit Alexa 488-conjugated secondary antibody (Invitrogen, Thermo Fisher Scientific, USA). The results were visualized by Leica TCS-SP8 confocal microscopy equipped with 10X, 20X, 40X and 100X objectives (Mannheim, Baden-Wuerttemberg, Germany).

***iTRAQ***

Two different plasmids (OE-TRIM65, OE-control) and two si reagents (siTRIM65, siControl) were transfected into KM12 cells in parallel. Cells were collected to prepare protein lysates, and western blotting was performed to confirm TRIM65 expression. Protein samples were then prepared and quantified; equal amounts of protein were used for subsequent protein digestion and iTRAQ labeling (“siTRIM65” was labeled with 114, “siCTL” was labeled with 115, “OE-CTL” was labeled with 116, and “OE-TRIM65” was labeled with 117). The labeled samples were combined and dried in vacuo, and the peptide mixture was subjected to high pH phase separation and then low pH nano-HPLC-MS/MS analysis. Data were acquired in a data-dependent mode using MASCOT 2.3 software and analyzed by MaxQuant (Version 1.5). Enrichment analysis for differential proteins were done in FunRich_V3 (18).

***Coimmunoprecipitation, in-Gel Tryptic Digestion and Mass Spectrometry (MS)***

Cell lysates were precleared by centrifugation and then incubated with the antibodies for FLAG (Sigma F1804) for two hours at 4℃ on a rotating wheel, according to the recommended protocol by the supplier. Then, the mixes were incubated together with the Sepharose-conjugated protein G magnetic beads (Thermo Fisher Scientific, Waltham, MA, USA) for 24 h at 4℃. After extensive washing, the beads were boiled to isolate the protein.

Protein samples were resuspended in loading buffer and reduced at 100°C for 5 min. The samples were separated by a 10% polyacrylamide gel. The gels were stained using Coomassie blue stain, and the target band was cut for in-gel tryptic digestion. Peptide samples were analyzed by nanospray-LC-MS/MS using an LTQ-Orbitrap Elite mass spectrometer (Thermo Fisher Scientific, San Jose, CA). Data were acquired in data-dependent mode using MASCOT 2.3 software. In more detail, whole cell lysates were immunoprecipitated with FLAG antibody (Sigma F1804) and then pulled down by IgG sepharose. Eluates were separated by SDS-PAGE and stained with Coomassie blue. IgG bands (two obvious bands, heavy and light chains) were cut and pulled together in one tube, and the rest were allocated evenly into 3 tubes. Thus, four tubes were used for each sample. After gel digestion and extraction, the IgG band was treated as one fraction and the rest as another fraction. Therefore, each sample contained 2 fractions subjected to LC-MS-MS. Raw data from 2 fractions were grouped together for database searching in PD2.2 (Human_refseq/ Q Exactive Plus/ ms1:Orbitrap-20 ppm/ ms2:Orbitrap-0.05 Da Phospho (ST),Phospho (Y),GlyGly (K),Oxidation (M),Acetyl (Protein N-term),DeStreak (C)).

***PLA***

Interaction between TRIM65 and ARHGAP35 was detected by using an *in situ* PLA kit (Sigma-Aldrich; Merck KGaA, Darmstadt, Germany) in the KM12 cell line. The ubiquitin-ARHGAP35 interaction was detected by PLA in KM12 48 h after transfection with *pCDNA-TRIM65* and *pCDNA-Empty* vector or TRIM65 and control siRNA (termed *siTRIM65* and *siCTL*). The PLA assay was followed according to the manufacturer's instructions. Briefly, PLAs were performed on fixed, permeabilized cells, with incubation of the primary antibodies at 4°C overnight. The antibodies were used at the following concentrations: anti-TRIM65 (1:50, H00201292-B01P, NOVUS), anti-ARHGAP35 (1:50, 26789-1-AP, Proteintech), anti-FLAG (1:50, F1804, Sigma), and anti-Ub (1:50, sc-8017, Santa Cruz). PLA minus and PLA plus probes (containing the secondary antibodies conjugated to oligonucleotides) were added and incubated for 1 h at 37℃. Ligase was used to join the two hybridized oligonucleotides into a closed circle. The DNA was then amplified (with rolling circle amplification), and detection of the amplicons was carried out using a Red Detection Kit for fluorescence. Cell nuclei were stained with DAPI. The results were visualized by TCS-SP8 confocal microscopy (Leica, Mannheim, Baden-Wuerttemberg, Germany), and the number of PLA signals was counted by a Duolink ImageTool (DUO90806-1EA, Sigma-Aldrich; Merck KGaA, Darmstadt, Germany).

***Ubiquitination Assay***

KM12 cells were transfected with the indicated *pCDNA-TRIM65* and *pCDNA-Empty* plasmids or TRIM65 siRNA (termed *siTRIM65)* with scramble control (*siCTL*). After 24 h, *pCDNA-His-UB* was transfected into the cells. The next day, the cells were treated with 25 µM MG132 (#s2619, Selleck Chemicals, HX, USA) for 4 h. The ubiquitinated proteins were pulled down with Ni-NTA magnetic agarose (#78605, Thermo Fisher Scientific, USA) following the manufacturer’s instructions. The protein complexes were then probed with anti-ARHGAP35 antibody to visualize the level of ubiquitination.

***Turnover Assay***

Cells were transfected with *pCDNA-TRIM65* and *pCDNA-Empty* plasmids and incubated in 5% (v/v) CO2 at 37°C for 24 h. Cycloheximide (CHX, MedChem Express) was then added to the media at a final concentration of 100 μg/ml for the indicated times. The cells were harvested, and ARHGAP35 protein levels were analyzed by immunoblotting.

***G-LISA Assay***

RhoA, Rac1, and Cdc42 were quantified using the G-LISA Rho Activation Assay Biochem Kit (Cytoskeleton, Inc.). After stimulation, the cells were washed twice with cold PBS and lysed using the lysis buffer provided with the kits for 15 min on ice. The lysates were centrifuged at 10,000 × g for 1 min at 4°C. Protein concentrations were determined. The G-LISA assay was performed and assessed following the manufacturer's protocol. Briefly, 120 µl of cell lysis buffer combined with 1.2 µl of the protease inhibitor cocktail and 120 µl of the binding buffer were mixed and used as a buffer blank, and a mixture of 24 µl of the RhoA control protein, 96 µl of the cell lysis buffer, and 120 µl of the binding buffer was used as a RhoA-positive control. KM12 lysates were adjusted to 1 mg/ml, and 90 µl of each lysate sample was mixed with 90 µl of the binding buffer. These samples, the buffer blank and the RhoA/Rac1/Cdc42-positive control (50 µl) were distributed to wells. Anti-RhoA/Rac1/Cdc42 monoclonal antibody was added as a primary antibody and detected using horseradish peroxidase, and the absorbance was measured at 490 nm.

***Statistical Analysis***

Chi-square tests and one-way ANOVA were used to assess differences in clinical variables between the CRC cohorts. Kaplan-Meier survival analyses were used to compare survival times among CRC patients based on TRIM65 expression; the log-rank test was used to generate *p* values. Cox proportional hazards regression analyses were used to assess the effect of clinical variables on patient survival. Univariate and multivariate analyses were used to assess the influence of clinical variables on survival. The *p* values and hazard ratios are indicated. Differences between groups were evaluated using a two-tailed t test or a Mann-Whitney U test. All statistical analyses were performed using SPSS 22.0 or GraphPad Prism 7. Paired samples were compared using a paired t test.

***Study approval***

A written informed consent for each patient about tissue sampling was obtained and the study was reviewed and approved by the Medical Ethics Committee of the Sixth Affiliated Hospital, Sun Yat-sen University. The animal studies were reviewed and approved by the Animal Care and Use Committee of SYSU.

**Plasmids and antibodies**

**Figure legends for supplemental figures**

**Figure S1.** TRM65 promotes the proliferation, migration and invasion of CRC Cells.

(A) qRT-PCR and (B) immunoblots of TRIM65 in *TRIM65-*KM12 and *shTRIM65*-KM12) in comparison with corresponding *Empty-*KM12 and *shCTL*-KM12 controls.

(C-D) Proliferation assay of HCT8 CRC cells conducted by using IncuCyte. HCT8 cells were stably transfected with *lenti-TRIM65* (termed *TRIM65-*HCT8) or *shTRIM65* vectors (termed *shTRIM65*-HCT8) in comparison with corresponding empty (*Empty-*HCT8)/control (*shCTL*-HCT8) vectors.

(E) Images and (F) statistics analysis of tumors isolated from subcutaneous xenografts in nude mice injected with *shTRIM65-KM12* cells or the *shCTL-KM12* control.

**Figure S2.** TRIM65 modulates Rho activity via ubiquitination of ARHGAP35.

(A) Representative DIC image of cells transfected with TRIM65 or empty plasmid.

(A) Top enrichment of biological pathways for differential genes between siCTL and siTRIM65 analyzed by iTRAQ experiment (Enrichment done in FunRich_V3).

(C) Immunoblots (upper panel) and correlation (lower panel) between the expression of TRIM65 and ARHGAP35 in 10 CRC cell lines.

(D) The mRNA expression level of TRIM65 and ARHGAP35 in several cell lines transfected with *siTRIM65* in comparison with *siCTL* controls.

(E) IF images of KM12 cells that were transiently transfected with *pcDNA-TRIM65* or empty control by using Alexa Fluor 488 phalloidin staining for the actin cytoskeletal fibers. Scale bar: 100 µm.

**Figure S3.** Representative Immunofluorescence (IF) images for various plasmid transfection.

1. IF result of KM12 transfected with pCDNA-Empty plasmid.

(B) IF result of KM12 transfected with pCDNA-TRIM65 plasmid

(C) IF result of KM12 transfected with pEGFP-N1-TRIM65 plasmid.

Scale bar: 25 μm. red: IHC with primary anti-TRIM65, green: GFP, blue: DAPI staining.
